# Potential application of *Staphylococcus devriesei* MS as a biosorbent agent for manganase, chromium, and cadmium heavy metals in contaminated water

**DOI:** 10.1038/s41598-025-91961-6

**Published:** 2025-03-21

**Authors:** Amany M. Shabaan, Marwa S. Embaby, Amany M. Reyad

**Affiliations:** 1 Chemistry Department, Biochemistry division, Faculty of Science, Fayoum University, Fayoum, Egypt; 2 Botany Department, Faculty of Science, Fayoum University, Fayoum, Egypt

**Keywords:** Heavy metals, Biosorption, *Staphylococcus devriesei*, SEM-EDX, FTIR, TEM, Biotechnology, Microbiology

## Abstract

This study identified one bacterial isolate as S*taphylococcus devriesei*, which is resistant to cadmium (Cd), manganese (Mn), and chromium (Cr) using 16S rRNA gene sequencing. Following that, the strain sequence was submitted to GenBank under accession number PQ013181. In this investigation, the biosorption potential of *Staphylococcus devriesei* was evaluated for the biosorption of chrmoium, cadmium, and manganese ions. The effects of pH, contact time, and initial concentration were examined in a batch-mode study. According to our findings, after 6 h at the ideal pH, *Staphylococcus devriesei*’s maximal biosorption capabilities of Cr and Cd were 98 and 81.2%, respectively. The maximum biosorption of Mn was 95.6% after 24 h at pH 6. SEM micrographs showed that, *Staphylococcus devriesei* were irregular and cracked with wrinkles on the surface after absorbing the studied Cr metal ions. It was observed that the alterations in cell size occurred when the bacterium was exposed to a dose of Mn and the aggregation of cells was seen. Bacterial cells treated with Cd exhibited irregularities, featuring depressions on their surfaces, and surface wrinkles. FTIR analysis showed obvious alterations in peak positions and intensities before and after the biosorption process. Energy dispersive X-ray analysis showed extra metal depositions on the treated cell surface compared to the control. At the ultrastructural level, TEM imaging demonstrates the involvement of extracellular and intracellular precipitates and accumulated metals on the cell walls. Thus, the results of this study indicated that *Staphylococcus devriesei* can effectively aid in the remediation of contaminated water with moderate to light levels of Cd, Cr, and Mn.

## Introduction

Industrial communities have significantly raised the levels of toxic heavy metals in the environment on a global basis. Almost all ecosystems are exposed to heavy metals through atmospheric, aquatic, and terrestrial transportation^1^. Serious issues with the environment, animal and human health result from their release into the ecosystem^2–4^. The deactivation of enzymes, blocking of active sites or functional groups of metabolically significant molecules, displacement or substitution of essential elements, and disruption of membrane integrity are just a few examples of how heavy metals can alter a variety of physiological processes at the cellular/molecular level and cause toxicity^5,6^.

Chromium, lead, cadmium, nickel, mercury, zinc, and copper can accumulate in amounts that are hazardous to living organisms in the soil and aquatic environment^7,8^. In both vertebrates and invertebrates, heavy metal toxicity causes oxidative stress, disrupts protein folding, and impairs physiological function^9,10^. Chromium is one of the most distributed elements in the earth’s crust, chromium has several oxidation states, ranging from Cr (0) (elemental chromium) to Cr (VI) (hexavalent chromium)^11,12^. Due to its relationship with other metals and salts including sodium and potassium chromates, dichromate, potassium and ammonium chrome alums, and other metals, chromium is among other metals established to be toxic. Additionally, it is found in elemental form in gases, plants, soil, animals, volcanic dust, and rocks^13^. The chromium (VI) is recognized to be very noxious and mutagenic and can cause cancer in animals and human beings^14^. Cadmium is a widespread health issue that, in some cases, may contribute to annual deaths. It affects several organs. Cancer and organ system toxicity, including damage to the skeletal, urinary, reproductive, cardiovascular, central and peripheral nervous, and respiratory systems, are caused by prolonged exposure to cadmium through the air, water, soil, and food^15^. Sources of exposure to cadmium are fossil fuels, iron and steel production, cement nonferrous metals production, waste incineration, smoking, fertilizers, etc^16^.

Manganese is one of the naturally occurring heavy metals which easily oxidized and chemically active^17^. It can form rust when reacts with water and iron, also dissolve in acids^18,19^. The primary hazardous side effects of manganese are extra-pyramidal side effects that closely resemble Parkinson’s syndrome symptoms^20^. The adverse effects are a result of its accumulation in particular basal ganglia structures and modification of the enzyme activity of dopaminergic neurons. Hepatotoxicity, cardiotoxicity, and an overall increase in neonatal mortality are other notable outcomes^21^.

A prominent method for removing these metals among the different techniques is biosorption employing various biomaterials, which is adaptable to metal concentrations and conditions and demonstrates an environmentally acceptable approach, also microbial biomass is frequently cited as having a higher biosorption capacity than other biosorbents for the removal of heavy metals^22–24^. Among multiple microorganisms, bacterial biomass can be produced simply and inexpensively, and it has been extensively studied for biosorption^25^.

The bacterial species studied in previous various studies include *Paraclostridium bifermentans* G3, *Pseudomonas* sp and *Ochrobactrum*^26–28^, *Staphylococcus xylosus*,* Microbacterium* sp. D2-2, *Bacillus* sp. C9-3, and *Lysinibacillus varians*^29–31^. The toxicity of Cd, Mn, and Cr has been demonstrated to be effectively reduced by a variety of microbial detoxification methods, including adsorption, bioreduction, and bioaccumulation^32,33^. Unfavorable circumstances can cause stress reactions that show distinctive alterations in bacterial cell shape and assembly. Examples of these conditions include exposure to poisonous metals, metalloids, and organics; extremely acidic or alkaline pH, and suboptimal temperatures^34,35^. Therefore, the present investigation was performed for the isolation and characterization of Cd, Cr, and Mn resistant bacterium from a heavy metal contaminated site for the biosorption process. Furthermore, morphological changes and biosorption mechanisms were examined using fourier-transform infrared spectroscopy (FTIR), scanning electron microscopy (SEM), and transmission electron microscopy (TEM).

## Materials and methods

### Chemicals and preparations

All the chemicals and reagents used are of analytical grade; different metal solution concentrations were prepared from cadmium standard solution (1000 mg/L) (Merck, Germany), chromium standard solution (1000 mg/L) (Merck, Germany), and manganese standard solution (1000 mg/L) (Merck, Germany). Tryptic Soya Broth and plate count agar also used to culture and isolate bacteria. Biosorption experiments were carried out in a double distilled aqueous solution of metal.

### Isolation and purification procedures

Sewage activated sludge was collected from the aeration tank of a wastewater treatment plant located at Fayoum, Egypt. The activated sludge was serially diluted with distilled water from dilution 10^− 1^ to 10^− 5^, 1.00 mL was taken and spread on plate count agar with different metal concentrations and incubated at 28ºC for 2 days. Finally, the most resistant isolate to the highest metal concentration was selected; morphologically distinct colonies were purified by repeated culturing on plate count agar plates. The purified target isolate was stored at − 80ºC for future experiments.

### Characterization and identification of the bacterial isolate

Gram staining was done to identify the strain for startup identification. Also, biochemical characterization was done to identify the isolated strain which included catalase test^36^, nitrate reduction test^37^, β-galactosidase test^38^, arginine dihydrolase, lysine decarboxylase & ornithine decarboxylase tests^39^, gelatinase test^40^, oxidase test^41^, amylase test^42^, urease test and sugar fermentation tests and other biochemical tests like hydrogen sulfide production test, indole test, and citrate test. For the identification of the isolated bacteria, the genomic DNA was extracted using standard bacterial procedures^43^. The PCR mixture was prepared as the following: 10 µL (10x) PCR buffer, 3 µL (50 mM) MgCl_2_, 1 µL (20 pmol/µL) of each primer, 1 µL (10 mM) Mixture of dNTPs, 0.5 µL (2.5U) Taq polymerase, 2 µL gross DNA extract, and volume completed by sterilized distilled H_2_O to 100 µL. Under the conditions set out below, PCR was performed for 35 cycles: denaturation stage at 90–94 °C for 40 s, the annealing step was controlled for 1 min at 55 °C, for the extension step, 72 °C for 2 min was adjusted, and the final expansion for 10 min at 72ºC . 10 µL from the products of PCR was added to 2 µL of DNA the gel containing 0.5 µg/mL ethidium bromide in the Tris–Borate-EDTA (TBE) buffer was then visualized using a UV transilluminator by the buffer loading and electrophoresis analysis on 0.7% horizontal agarose (60 min at 15 V/cm, ). The sequencing of the amplified fragments was completed at GATC Biotech, Constance, Germany. DNA sequence were aligned at NCBI database (www.ncbi.nlm.nlh.gov). The phylogenetic tree was established using a neighbor-joining technique using TREEVIEW software (1.6.6) derived from gene sequences of 16 S rRNA of some phylogenetic close strains to the isolated strain. The sequence was submitted to GenBank NCBI database to have an accession number^44^.

### Biomass preparation

The bacterium was cultivated in Tryptic soya broth for 2–3 days at 28ºC . The cells were harvested by centrifugation at 4,000 rpm for 20 min. The collected biomass was washed three times with sterilized tap water and then used in the batch experiment.

### Biosorption experiment

The study on biosorption was conducted with 0.04 g/L living bacterial biomass in a 50 mL metal-containing solution. The pH was adjusted to 6, 6,and 7 for Cr, Mn, and Cd, respectively, and the samples were shaken for 6, 6, and 24 h for Cr, Cd, and Mn, respectively. Biosorption was independently studied using the concentrations of 5 mg/L of each metal ion. After the reaction, the mixture was centrifuged for 20 min at 4,000 rpm. ICP (Perkin Elmer optical emission spectrometer [OES], Optima 5300 DV) was used to determine the concentrations of Cr, Cd, and Mn in the solution. Metal uptake on bacterial biomass (%) and the biosorption capacity (mg/g)^45^ were calculated using the following equations:

$$\% = \frac{{\left( {C_{{\text{o}}} - C_{{\text{e}}} } \right)}}{{C_{{\text{o}}} }}*100$$

$$q_{{\text{e}}} = \frac{{\left( {C_{{\text{o}}} - C_{{\text{e}}} } \right)V}}{m}$$

Where ${\text{C}}_{{\text{o}}}$ and C_e_ are the initial and final concentrations of the metal ions in mg/L, respectively, *V* represents the volume of the solution (L), and *m* shows the weight of the biomass (g).

### SEM-EDX, TEM, and FTIR analyses

SEM, TEM, and FTIR analyses of the bacterial biomass were performed before and after loading with metal solutions. The FTIR spectroscopy technique was employed to expose the biosorption binding mechanism of Cd (II), Cr (III), and Mn (II) by bacterial biomass. One important characteristic of bacterial biomass is the functional groups present on the surface; FTIR analysis was carried out to investigate the shift in functional groups following metal loading. This technique was used for providing a qualitative description^46^.

The characterization of bacterial biomass by scanning electron microscopy (SEM) provides topographical and elemental information about the solids with a virtually enormous depth of field, allowing various specimen parts to keep in focus at a time. High resolution in SEM also enables greater magnification for closely spaced materials. In addition to its capability to create an actual clear image, it is also helpful for discovering the topographical aspects of bacterial biomass before and after metal loading^47^. EDX elemental analysis was provided to check the adherence of metals on bacterial cell surface. JEOL JEM-1400 transmission electron microscopy (TEM) was performed to confirm the biosorbent capability of the bacterial strain to the various metals observing the accumulation the metals on its cell wall and also inside cells. Prior to its analysis, the biomass sample was kept in contact with each metal for the suitable optimum time (six hours contact time for Cr & Cd and 24 h for Mn). Preparation of the sample was performed according to the protocol described by Tsezos et el^48^.

### Analytical applications in drinking raw water sources in Fayoum, Egypt

To check the applicability of the bacterial biomass (*Staphylococcus devriesei* MS) in the biosorption of metal, a water sample was collected from the intake of New Azab Plant located in Fayoum Governorate, Egypt and the recommended procedure for biosorption of the studied metals was applied. The water sample was collected in a 2-liter polyethylene bottle, filtered through a 0.45 μm glass membrane filter. The water samples spiked to 5 mg/L for each studied metal ion, then adjusted to the optimum conditions. Experiments were carried out using 0.04 g of bacterial biomass under study and 50 mL of spiked water samples, then the water samples were equilibrated for the optimum hours for each metal in a mechanical shaker. The samples were then centrifuged, filtered, and analyzed for Cr (III), Cd (II), and Mn (II) content using ICP-OES by standard methods.

### Statistical analysis

The mean ± standard deviation (SD) was determined for triplicate experiments of biosorption of metals. Statistical analysis was performed using R software^49^ (RCoreTeam, 2023). A P value < 0.05 is considered statistically significant.

## Results

### Bacterial characterization and identification

**Fig. 1:**
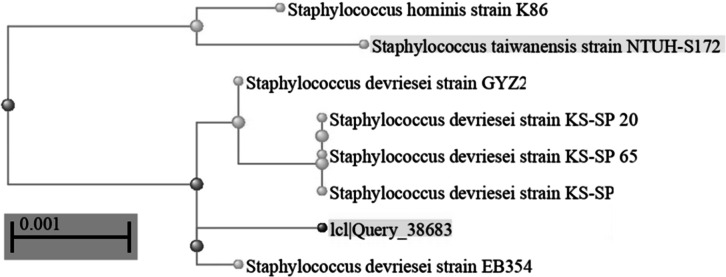
16 S rRNA gene sequence-based neighbor-joining phylogenetic tree, shows the position of our heavy metal resistant strain among members of the related species.

The most metal-resistant bacterial isolate was isolated, it tolerated to 50 mg/L. As shown in Table 1, a range of morphological and biochemical tests were performed to provide a thorough understanding of the morphological and biochemical characteristics of the bacterial isolate. Gram-positive, non-motile cocci and does not form spores. The bacterial isolate expressed negative results to β-galactosidase, ornithine decarboxylase, arginine dihydrolase, oxidase, gelatinase, and H_2_S production. In the meantime, the tests for lysine decarboxylase, catalase, urease, amylase, nitrate production, citrate utilization, and indole production yielded positive findings (Table 1). Concurrently, the bacterial isolate demonstrated the capacity to use inositol, sorbitol, ribose, and raffinose as carbon sources. To compare the DNA sequences to unidentified sequences, the National Center for Biotechnology Knowledge’s Blastx program (BLAST) was utilized. It can be clearly seen that this bacterium was included in the genus *Staphylococcus* and is closely related to the species *devriesei.* It showed the highest sequence similarities with *Staphylococcus devriesei* strain EB354. The phylogenetic tree was established and demonstrated in Fig. (1). The strain sequence was submitted to GenBank and had accession number **PQ013181**.

**Table 1:** Bacterial characterization.

Reaction	Result	Reaction	Result
Morphological characters		Fermentation of sugars	
Gram staining	Positive	D-Glucose	Positive
Motility	Non- motile	Sucrose	Positive
Cell shape	cocci	D-Mannitol	Positive
Endospore formation	Negative	Inositole	Negative
Biochemical characters		Sorbitol	Negative
Enzyme profile		D-Fructose	Positive
β-galactosidase	Negative	Lactose	Positive
Arginine dihydrolase	Negative	Amygdalin	Negative
Lysine decarboxylase	Positive	Arabinose	Negative
Ornithine decarboxylase	Negative	Maltose	Positive
Urease	Positive	D- Mannose	Positive
Gelatinase	Negative	Trihalose	Positive
Catalase	Positive	Rafinose	Negative
Amylase	Positive	D-Ribose	Negative
Oxidase	Negative	Cellobiose	Negative
Nitrate reduction	Positive	Other tests	
		Citrate utilization	Positive
		H_2_S production	Negative
		Indole production	Negative

### Biosorbents characteristics and the influence on the metal binding process

#### Scanning electron microscope (SEM) and energy-dispersive X-ray (EDX)

**Fig. 2:**
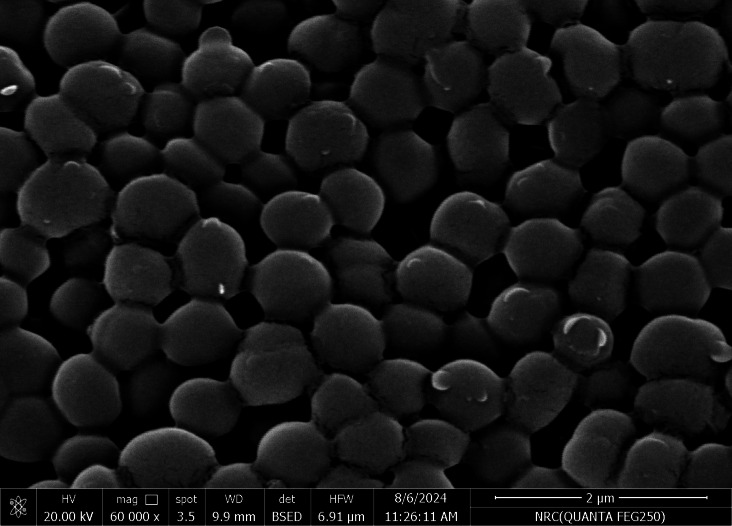
SEM micro-image shows the control cells of S*taphylococcus devriesei* MS (rounded normal cells with smooth surfaces)

**Fig. 3:**
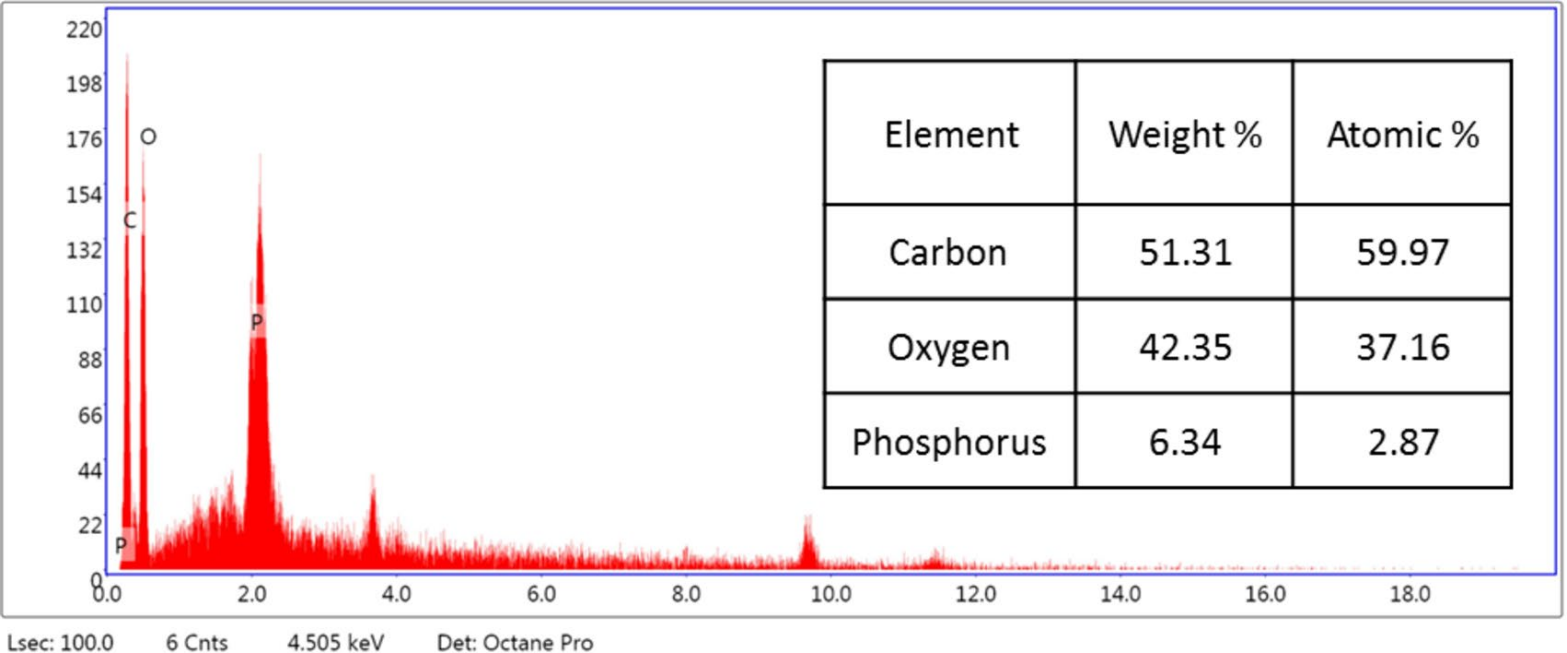
EDX analysis shows the normal cells of S*taphylococcus devriesei* MS

**Fig. 4:**
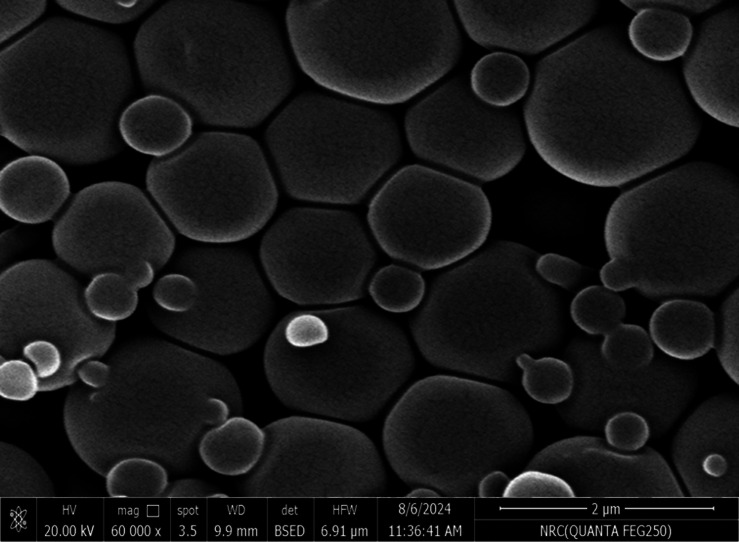
SEM micro-image shows the Mn biosorbent cells of S*taphylococcus devriesei* MS (some cells became smaller in size, more brilliant, and aggregated)

**Fig. 5:**
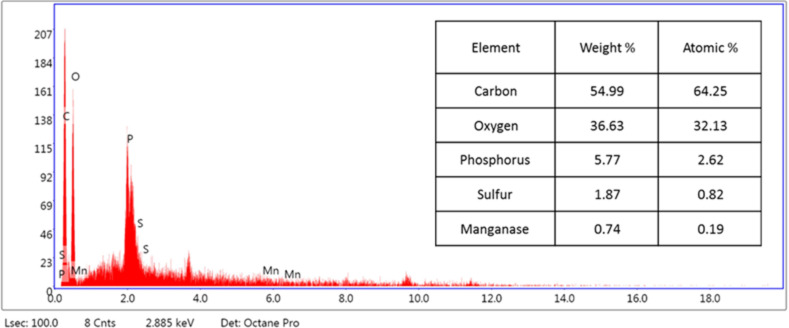
EDX analysis shows the extra Mn metals among the elemental constituents of Mn biosorbent cells of S*taphylococcus devriesei* MS

SEM was used in our investigation to observe how the biosorption of metal ions on the bacterial cell surface would alter the cell-surface morphology, while The EDX analysis was used to determine the insertion of the studied metal ions (Cd, Cr, and Mn) into the cell wall of *Staphylococcus devriesei* strain MS after the biosorption process. Figures 2 and 3 demonstrate the appearance of the control cells that are rounded and of entire margins of typical elemental analysis. Figures 4 and 5 show the alteration in bacterial cell size that was treated with Mn, cells became more closer and compact with extra Mn metals on its surface. Figures , 6, 7, 8, and 9 demonstrated the cells treated with Cr and Cd, cells became aggregate, exhibited irregularities, featuring depressions on their surfaces and surface wrinkles with extra Cr and Cd elements on their surfaces. TEM imaging confirms the metal biosorbent ability of the bacterial strain cells (Figs. 10). A TEM image of the Cr-treated cells reveals a black layer on the cell wall and dense, dark precipitates inside the cells, demonstrating the bacterial strain cells’ strong capacity for biosorption. Outside the cells, there were some dark precipitates visible. The cells treated with Mn exhibit aggregation, including the production of biofilms similar appearance. A black layer surrounds the aggregation, and the dark layers around each cell show how effective the cells are at biosorption. Inside the cells, dark precipitates were seen. The black deposit on the cell wall of the Cd-treated cells and several black precipitates were noticed outside the cells indicating the bacterial strain’s capacity for biosorption.

**Fig. 6:**
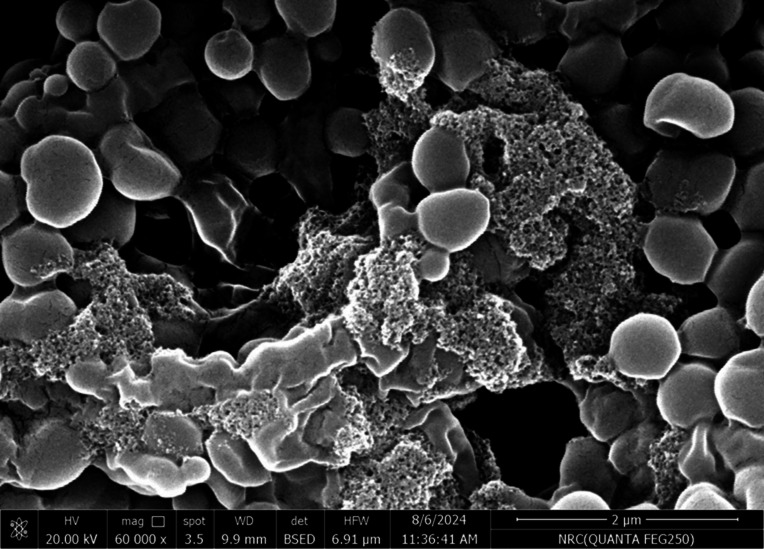
SEM micro-image shows the Cr biosorbent cells of *Staphylococcus devriesei*  MS (some cells have become more shrunken and their surface is not completely round, brilliant, and some deposits and clusters are found adjacent with bacterial cells)

**Fig. 7:**
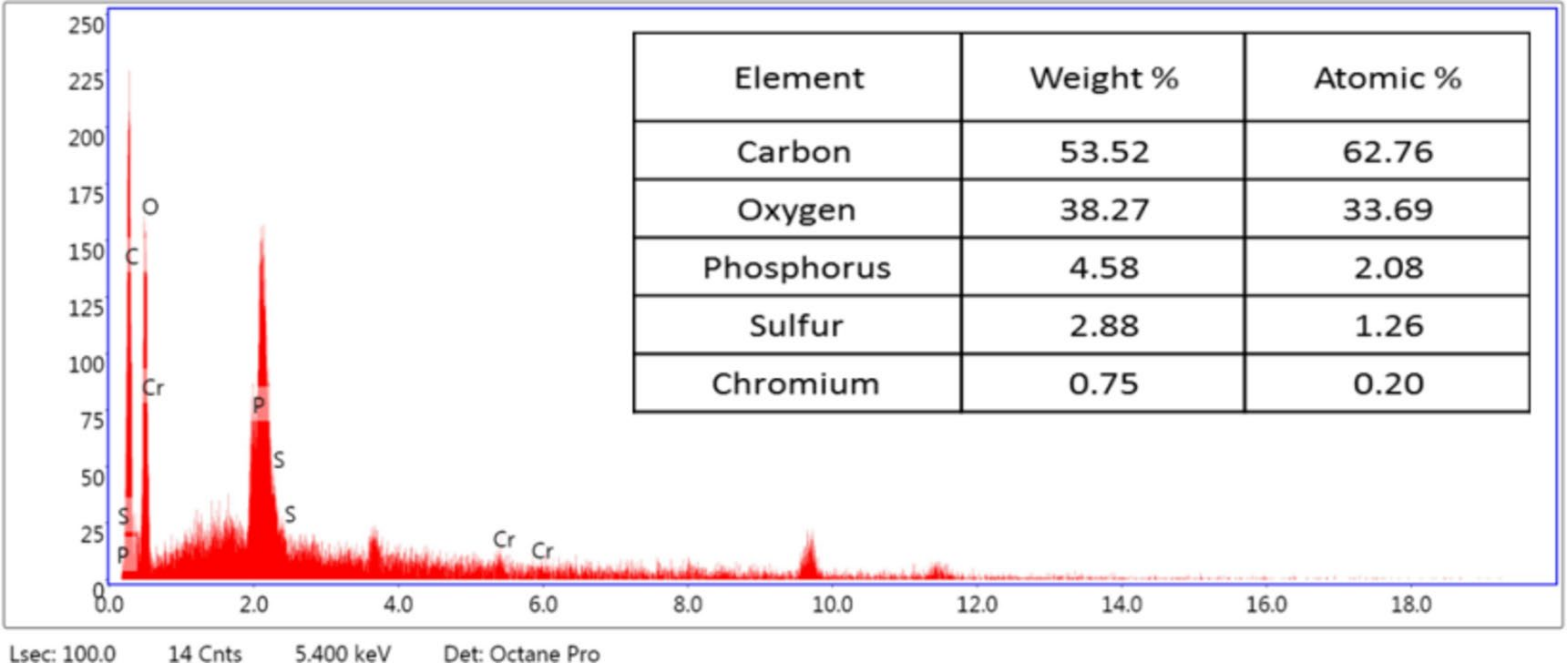
EDX analysis shows the extra Cr metals among the elemental constituents of Cr biosorbent cells of *Staphylococcus devriesei* MS

**Fig. 8:**
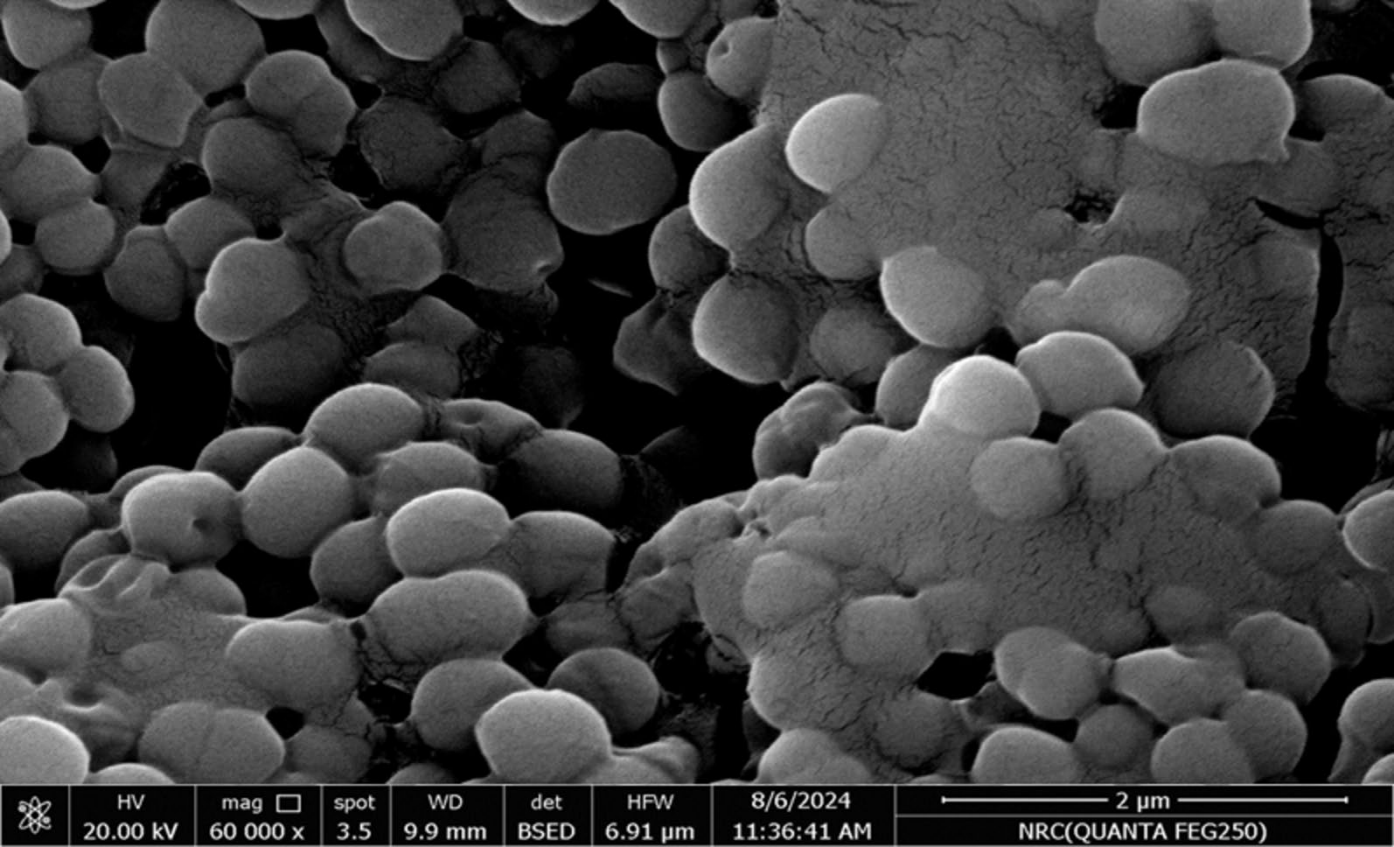
SEM micro-image shows the Cd biosorbent cells of *Staphylococcus devriesei* MS (some cells have notches and depressions on their surface and some deposits and clusters are found adjacent with bacterial cells)

**Fig. 9:**
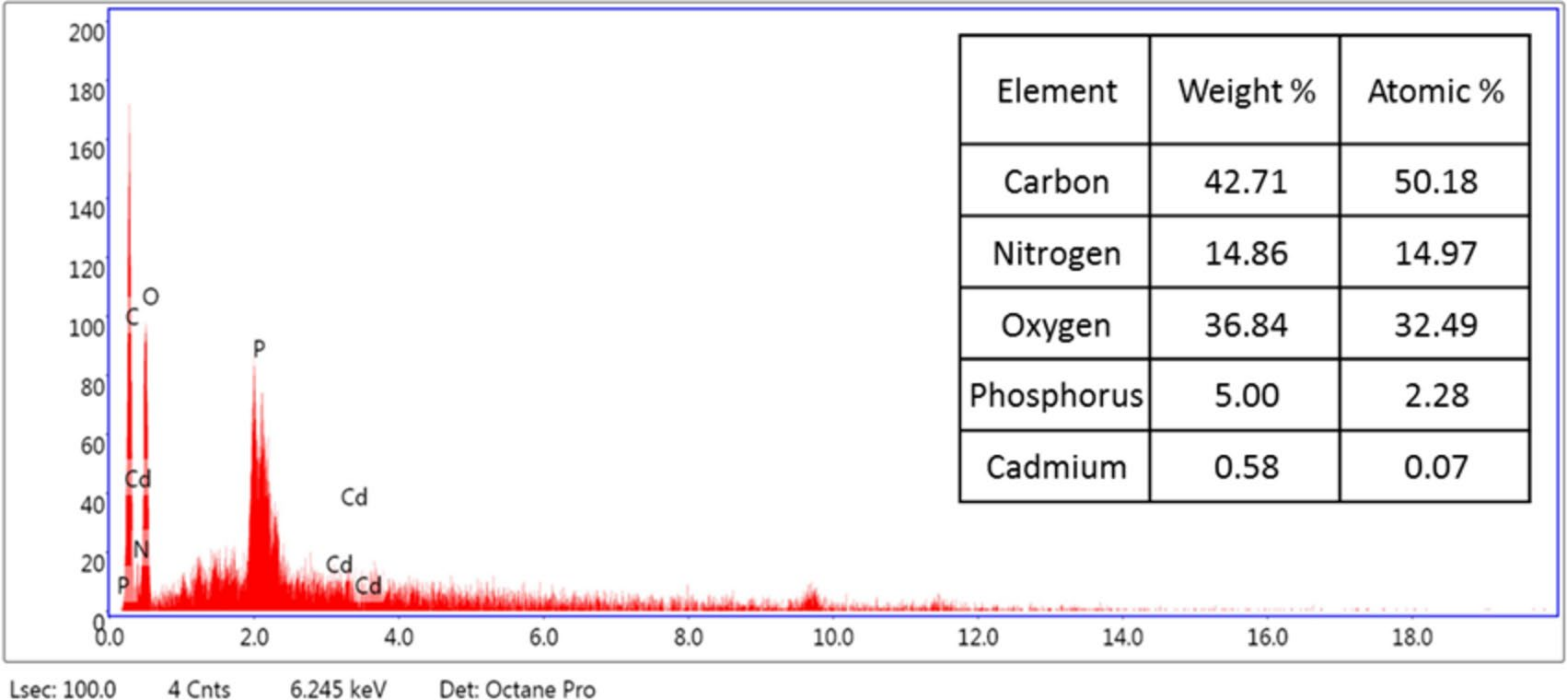
EDX analysis shows the extra Cd metals among the elemental constituents of Cd biosorbent cells of *Staphylococcus devriesei* MS

#### Cell surface change by FTIR analysis

The interactions between the functional groups on the active sites of the bacterial cell and the metal ions were demonstrated by FTIR analysis of the biomass and the biomass loaded with metal ions after biosorption process. Obvious alterations in peak position and intensity were observed before and after biosorption of metal ions (Fig. 11). The FTIR results suggest that the *Staphylococcus devriesei* strain MS is useful for the biosorption of Cr, Cd and Mn ions; FTIR analysis of the studied isolate confirms that the functional groups involved in the biosorption process and metal removal may have hydroxyl, alkyl, amide, halogen, phosphoryl, and phosphoric acid groups.

**Fig. 10:**
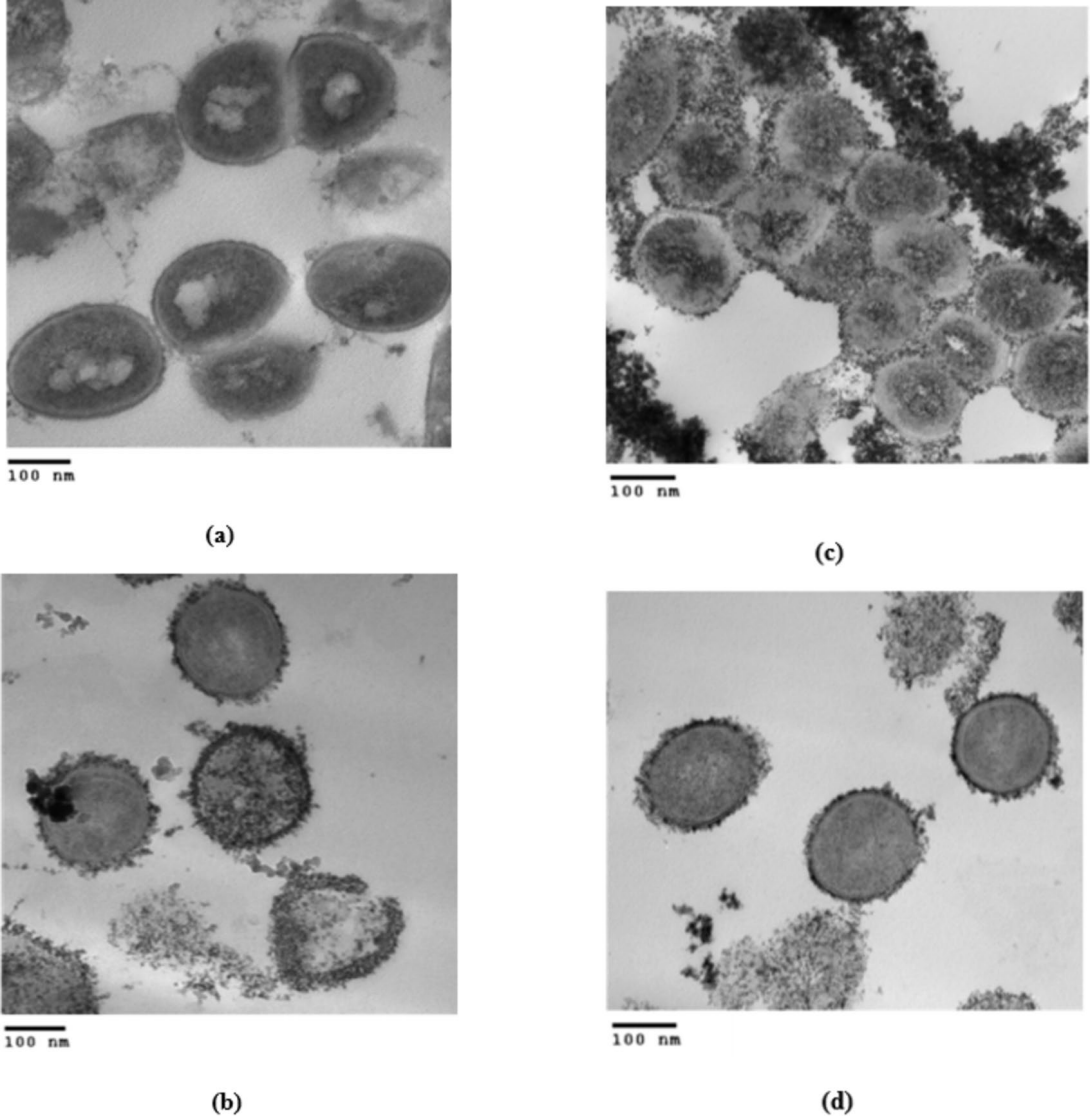
Transmission electron micrographs with HV = 80.0 KV and magnification = 60000X, (a) untreated control cells, (b) the bacterial cells treated with Cr for 6 h, (c) the bacterial cells treated with Mn for 24 h, (d) the bacterial cells treated with Cd for 6 h. (a) Untreated control bacterial cells. (b) TEM micrograph of the Cr treated cells shows the dark layer on cell wall and dense dark precipitates inside the cells indicating the highly biosorbent capability of the bacterial strain cells. Some dark precipitates were observed outside the cells. (c) TEM micrograph of the Mn treated cells shows their aggregation such as a biofilm formation. The aggregation is encircled by a black layer, and the dark layers surrounding each cell indicationg the high biosorption effeeciency of the cells. Dark precipitates were observed inside cells. (d) TEM micrograph of the Cd treated cells shows the dark layer on cell wall indicating the biosorbent capability of the bacterial strain cells. Some dark precipitates were observed outside the cells

In our results, the maximum absorption peak of the hydroxyl group (O-H) and amino group (N-H) is observed to be shifted after biosorption from 3,311.78 cm^− 1^ to 3,292.49 cm^− 1^. Also, the absorption peak of the carboxylic acid (C = O) peak at 1,647.21 cm^− 1^ was widened and moved to 1645.28 cm^− 1^ after biosorption in biomass loaded with chromium. The absorption peak at 1448.54 cm^− 1^ which is attributed to N-H vibrations, the peak moved to 1489.05 cm^− 1^ in biomass loaded with manganese. The absorption peak of the phosphoryl (P = O stretching vibration) at 1,404 cm^− 1^ was moved to 1,400 cm^− 1^ after biosorption. In our FTIR spectra, the absorption peak at 1,238 cm^− 1^ which attributed to the asymmetric stretching vibration of P = O of the phosphate group moved to 1242 cm^− 1^ after biosorption, also the peak became weaker in biomass loaded with (chromium & manganese) and also the absorption peak become widening in biomass loaded with cadmium. Furthermore, the absorption peak at 1,072 cm^− 1^ which was caused by P = O symmetric stretching vibration was widening, also the peak shifted after adsorption to 1,068 cm^− 1^ in biomass loaded with cadmium and manganese, and moved to 1,049 cm^− 1^ in biomass loaded with chromium. Also, the IR absorption band at 532.35 cm^− 1^ which was attributed to a strong stretching vibration of halogen linked to the ligand moved to 536.21 cm^− 1^ & 538.14 cm^− 1^ in biomass loaded by cadmium and manganese, respectively.

### Batch experiments

#### Effect of pH

The values of pH were adjusted for the investigation between 4 and 7, and the results for the percent of removal (% R) were determined (Table 2). As the pH increased, it was found that the percent removal for all three elements increased dramatically, as shown in Fig. (12)). It was discovered that the removal percentage values for Cr (III) and Mn (II) at pH 6 were 91.6% and 80.28%, respectively, and that the removal percentage value for Cd (II) at pH 7 was 79.6%. At low pH (*<* 5.0), the biosorption capacity for all metal ions is very low, due to a large quantity of H^+^ competing with metal ions at sorption sites, this causes repulsion of the metal ions from the bacterial cell which makes adsorption unfavorable. The strong positive correlations (e.g., between pH and metal removal %) indicating that increases in pH may enhance the removal efficiency of Mn, Cr, and Cd. The correlation coefficients (0.91 and 0.94) indicate a strong relationship. The significant correlation between Cr and Cd removal (0.97) indicate that these two metals may behave similarly under the tested conditions (Fig. 12).

**Fig. 11:**
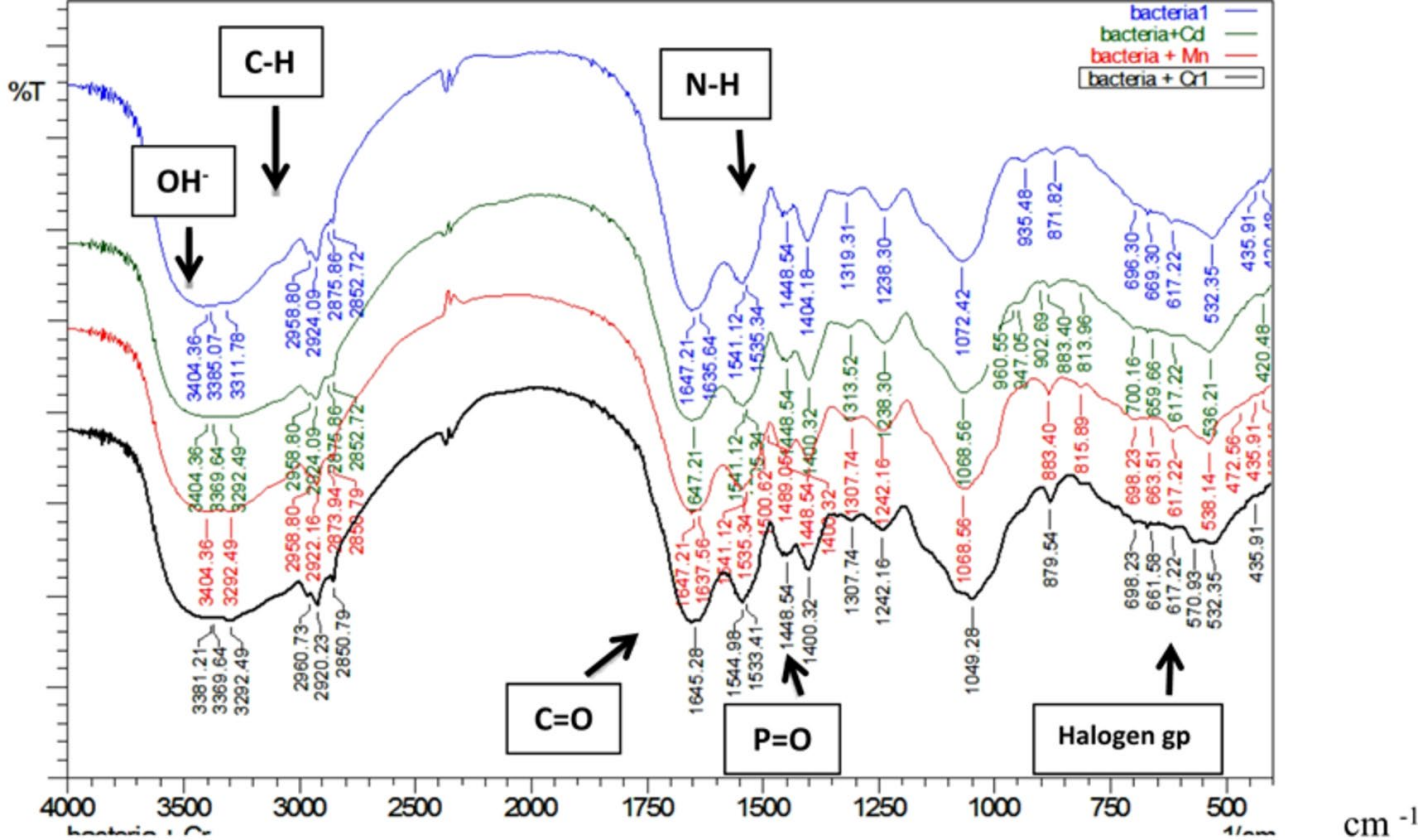
Infrared spectra of bacterial biomass and bacterial biomass loaded with metals

**Table 2:** Effect of “pH” on the absorption of Mn (II), Cr (III), and Cd (II) by *Staphylococcus devriesei* strain MS

pH	Mn^+ 2^ *R*% ± SD	Cr^+ 3^*R*% ± SD	Cd^+ 2^ *R*% ± SD
4	52.6 ± 0.721	44 ± 1.732	71.6 ± 0.611
5	67.2 ± 0.702	50 ± 1.137	71.2 ± 0.611
6	80.28 ± 0.255	91.6 ± 0.230	77.4 ± 0.6
7	68.2 ± 1.113	89.6 ± 0.346	79.6 ± 0.416

#### Effect of contact time

One of the fundamental elements of the biosorption process is contact time. The effect of contact time on the biosorption of Cr (III), Cd (II), and Mn (II) ions from solution was examined at various intervals between 2 and 36 h. Fig.13 Revealed the critical role of contact time in metal removal, with a notable negative correlation with Cd^+ 2^ removal (−0.52). This suggests that longer treatment times improve removal efficiency for certain metals while potentially hindering others. The negative correlations observed for Mn^+ 2^ and Cr^+^^3^removal with time (e.g., −0.75 and −0.33) might imply that as time increases, conditions may become less favorable for these specific removals. Mn uptake increases with a rise in contact time up to 24 h. The results are shown in Table 3, from which it is clear that at 6 h. of equilibration was sufficient for microbial biomass for maximum removal of Cr (III) and Cd (II). The percent removal values at maximum biosorption were found to be 91.6 and 79.6%, respectively, at contact times of six hours . For Mn (II), the percent removal value at maximum biosorption was found to be 80.28% at contact times of twenty-four hours.

**Fig. 12:**
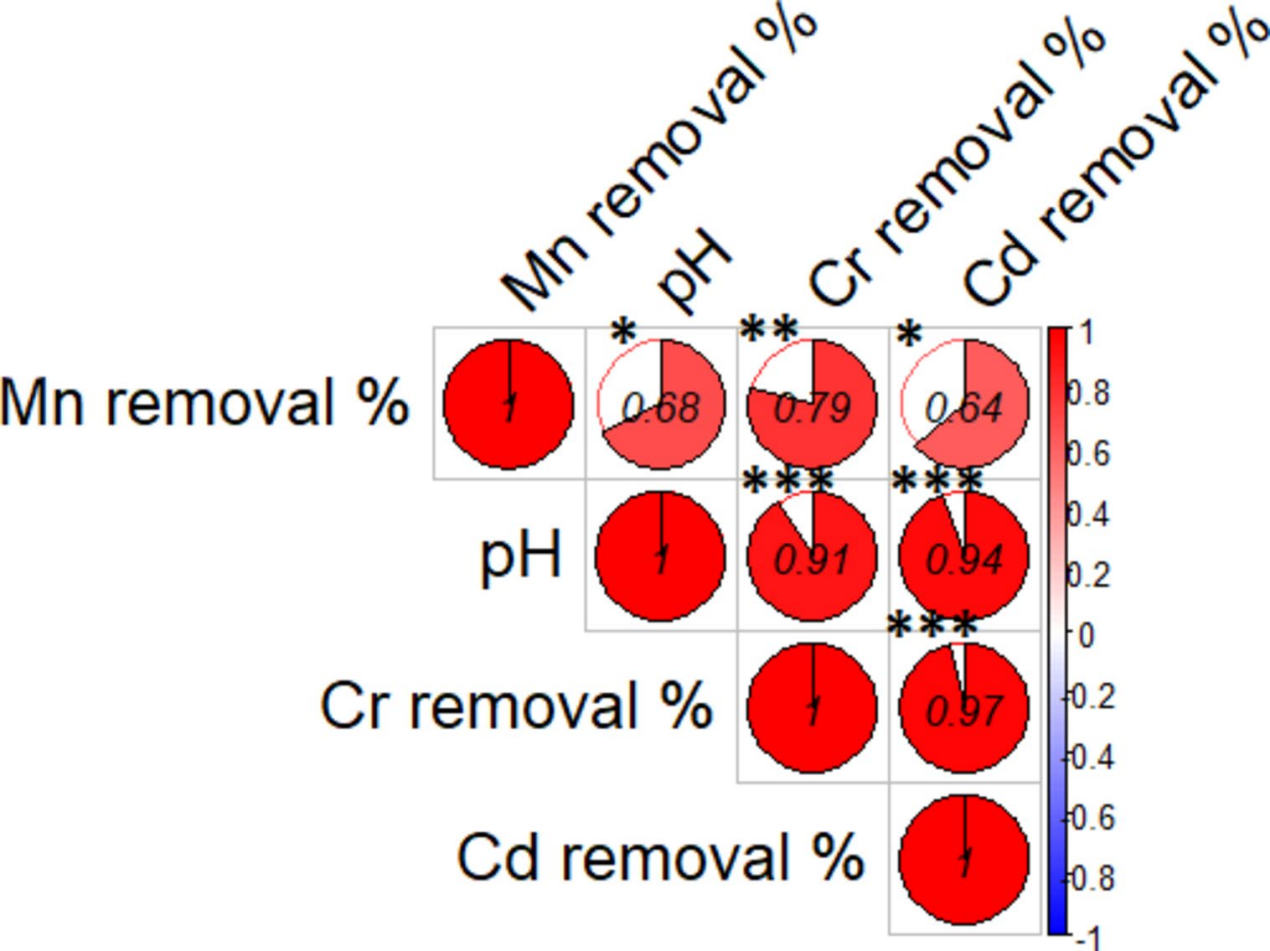
The correlations between pH and metal removal %. Asterisks denote significance levels, *refer to  *p* < 0.05, **refer to *p* < 0.01, ***refer to *p* < 0.001

**Table 3:** Effect of “contact time” on the absorption of Mn (II), cr (III), and cd (II) by *Staphylococcus devriesei* strain MS

Contact time (hr)	Mn^+ 2^ *R*% ± SD	Cr^+ 3^ *R*% ± SD	Cd^+ 2^ *R*% ± SD
2	77.2 ± 0.174	86.8 ± 0.150	77 ± 0.271
6	77.6 ± 0.20	91.6 ± 0.227	79.6 ± 0.499
12	79.4 ± 0.529	86.4 ± 0.041	79.4 ± 0.215
24	80.28 ± 0.266	57 ± 0.291	75.6 ± 0.094
36	73.4 ± 0.529	50 ± 0.220	75.1 ± 0.145

#### Effect of initial metal ion concentration

Fig. 14 shows the strong negative correlations involving concentration (e.g., −0.97 for Cd^+ 2^, −0.99 for Cr,^+3^ and −1 for Mn^+ 2^) suggesting that higher concentrations of these metals are detrimental to their removal efficiencies. The experimental results were obtained using various ion concentrations between 2 and 20 mg/L for the biosorption of Cr (III), Cd (II), and Mn (II) onto bacterial biomass. The maximum absorption of heavy metals reached at   2 mg/L and then gradually decreased (steady state) as the initial metal concentration increased. When the concentrations exceeded more than 10 mg/L, slightly reduced absorption capacity was observed as shown in Table 4.

**Fig. 13:**
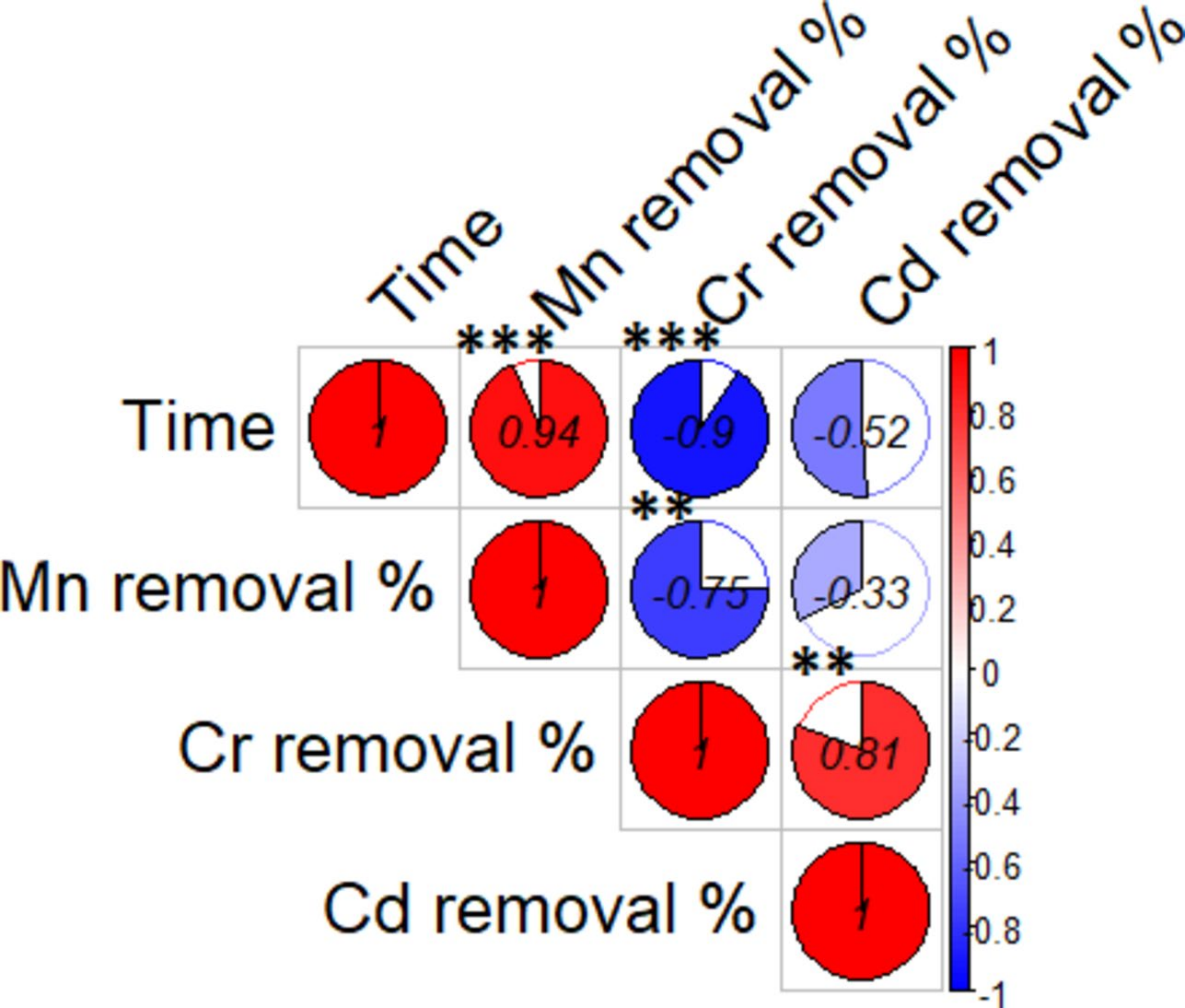
The correlations between the contact timeand the metal removal %. Asterisks denote significance levels, *refer to *p* < 0.05, **refer to *p* < 0.01, ***refer to *p* < 0.001

**Table 4:** Effect of “metal concentration” on the absorption of Mn (II), cr (III), and cd (II) by *Staphylococcus devriesei* strain MS

Metal concentration (mg/L)	Mn *R*%± SD	Cd *R*% ± SD	Cr *R*% ± SD
2	91 ± 0.05	96 ± 0.05	98 ± 0.05
5	80.28 ± 0.225	79.6 ± 0.416	91.6 ± 0.23
10	67.8 ± 0.011	77.9 ± 0.435	81.5 ± 0.23
20	32.98 ± 0.455	35.15 ± 0.332	40.065 ± 0.225

### Analytical applications in drinking raw water sources in Fayoum, Egypt

A good agreement between the added and measured analyte ions was established, as evidenced by the final results displayed in (Tables 5 and 6). The three replicates’ recovery and relative standard deviation (RSD %) were calculated both before and after biosorption. The results given in Tables 5 and 6 demonstrate that the results of the spiked samples showed high recovery percentages before and after biosorption. In Table 5, the samples spiked with 5 mg/L of standard solution of the tested metal ions, adjusted to the optimum pH for each metal then the metal concentration was measured in each prepared spiked sample before biosorption, the spiked sample solutions of Cr (III), Cd (II), and Mn (II), the recovery percentages are 98.4, 98.2, and 98.0%, respectively. While in Table 6, under optimal conditions, the biosorption process with *Staphylococcus devriesei* strain MS resulted in a removal percentage of 98.0, 81.2, and 95.6% for the metals Cr (III), Cd (II), and Mn (II), respectively.

**Table 5:** Analysis of prepared spiked water samples shows the metal recovery% (before biosorption), (*n* = 3).

Metal Ions	Without spiking(mg/L)	Spiked (mg/L)	Recovery%	RSD%
Cr^3+^	**0.0026**	5	**98.4**	**1.66**
Cd^2+^	**0.0004**	5	**98.2**	**1.73**
Mn^2+^	**0.0476**	5	**98.0**	**1.73**

^1^*SD* standard deviation.

**Table 6:** Analysis of prepared spiked water samples after biosorption by *Staphylococcus devriesei* strain MS, (*n* = 3).

Metal Ions	Without spiking(mg/L)	Spiked (mg/L)	Removal%	RSD%
Cr^3+^	**0.0026**	5	**98.0**	**1.98**
Cd^2+^	**0.0004**	5	**81.2**	**4.21**
Mn^2+^	**0.0476**	5	**95.6**	**10.14**

^1^*SD* standard deviation.

## Discussion

All over the world, the concentrations of hazardous heavy metals in the environment have increased dramatically due to industrial communities^50^. Heavy metals are present in almost every ecosystem due to terrestrial, marine, and atmospheric transportation^1^. Their discharge into the ecosystem causes major problems for the ecology, animal health, and human health^2^. Heavy metals can change many physiological functions at the cellular/molecular level and cause toxicity. Some examples include deactivating enzymes, blocking the active sites or functional groups of metabolically important molecules, replacing or displacing the necessary elements, and adversely affecting the integrity of the membrane^5,6^. Among the many approaches, biosorption using different biomaterials is a popular way to remove heavy metals because it can be adjusted to metal concentrations and conditions and shows an environmentally acceptable approach. Microbial biomass is also widely recognized as having a higher biosorption capacity than other biosorbents for heavy metal removal^22–24^. In the present study, it can be clearly seen that the isolated bacterium was included in the genus *Staphylococcus* and closely related to the species *devriesei.* It showed the highest sequence similarities with *Staphylococcus devriesei* strain EB354. The strain sequence was submitted to GenBank and had accession number PQ013181. The pH values play an important role in biosorption process; an appropriate pH value can not only improve the uptake efficiency of metal but also depress the matrix interference. Among all other parameters, the pH of the solution has been found to be the most important factor that affects the process of biosorption, it must be properly optimized because it directly affects adsorption capability^6^. The pH has a significant impact on the solution chemistry of the metals, the activity of functional groups on the cell wall (such as carboxylate, phosphate, and amino groups), and the competition amongst metallic ions for the binding site^51^. Our study results showed that at low pH (*<* 5.0), the biosorption capacity for all metal ions is very low because a large quantity of hydrogen ion (H^+^) competes with metal ions at sorption sites; this causes repulsion of the metal ions from the bacterial cell which makes adsorption unfavorable^52^. The presence of OH^−^ enhances the deprotonation reaction, which lowers the positive charge density on the adsorbent and facilitates a larger metal uptake. As pH rises, more negatively charged cells become available^53^. Many studies have previously investigated that for pH > 7. The metal ions in the solution precipitated due to the formation of hydroxides preventing the contact of the metal with the bacterial biomass^52,54^. So the investigation of the biosorption process for a basic range of pH (< 7) was not done. In our study, the rate of Cr (III) biosorption increases from 44 to 91.6% when pH is varied from 4 to 6, and the rate of Cd (II) biosorption increases from 71.6 to 79.6% when pH is varied from 4 to 7. While the rate of Mn (II) biosorption increases from 52.6 to 80.28% when pH is varied from 4 to 6. Therefore, pH 7 was found to be the optimum value for Cd (II) biosorption and pH 6 was found to be the optimum value for Cr (II) & Mn (II) biosorption. As revealed in the present study, the interaction between metal ions and the adsorbent sites is significantly dependent on the contact time; the biosorbent can exhibit its full biosorption capacity by extending the contact time in an experimental setup. Once the biosorbent has reached its maximum biosorption capacity at the optimum conditions, wherein the binding sites of the biosorbent have become completely saturated, any attempt to increase the duration of contact between the biosorbent and the metal ions would be futile and ineffective; our results have high agreement with^55,56^. It was observed that as the duration of biosorption increased the rate of metal ions biosorption onto bacterial biomass also increased. It took nearly 6 h to achieve complete saturation of the active sites of the studied bacteria for Cr (III) & Cd (II), while the duration of maximum biosorption for Mn (II) took 24 h. Through a combination of factors, including the availability of particular surface functional groups and their capacity to bind metal ions (particularly at high concentrations), the initial heavy metal concentration can affect the efficiency of metal removal^57^. The recent investigation revealed that the increase in metal concentration to surface area ratio is responsible for lower biosorption at higher metal concentrations. Higher metal concentrations result in a lower metal uptake because, for a biosorbent with a fixed concentration, the biosorbent sites are limited by the increasing concentration of the metal ions, similar results were obtained by^58,59^. To overcome the mass transfer resistance for metal ion transport between the solution and the biomass surface, the initial concentration serves as a driving force^57^. In our findings, the maximum absorption of heavy metal ions attained at 2 mg/L and then steadily declined (steady state) as the initial metal concentration increased, when the concentrations exceeded more than 10 mg/L. Slightly reduced absorption capacity was observed because of the saturation of absorption sites and the high number of metal ions competing for the readily available binding sites in the biosorbent^57^. Our results demonstrated that as the initial metal ion concentration increased, the absorption capacity abruptly increased and lower amounts of Cr (III), Cd (II), and Mn (II) were eliminated by the bacterial biomass. This behavior might be clarified by the fact that the bacteria become stressed due to the high concentration of heavy metals.

Using FTIR analysis, our spectra showed obvious alterations in peak position and intensity before and after biosorption of metal ions. The functional groups located on the active sites of bacterial cells and the physiochemical factors of the solution play a significant role in the biosorption of metal ions by the microbial biomass. Therefore, FTIR analysis of the biomass was performed in order to better understand the different kinds of functional groups participated in the biosorption process^51^. In the current investigation, peak shifting observed following the biosorption process demonstrated that functional groups might be involved in the biosorption of metal ions. Most biosorption investigations use FTIR specifically to check whether specific surface functional groups are present in the structure of biosorbents, though it is also possible to further investigate how these groups affect the metal binding process. On the basis of FTIR results, several research groups have offered helpful discussions about the metal binding mechanism on functional groups^60–62^. In our work, by comparing the infrared spectra of *Staphylococcus devriesei* strain MS before and after absorbing of metals, it can be seen that the maximum absorption peak of the hydroxyl group (O-H) and amino group (N-H) is broadened, and the peak is observed to be shifted after biosorption of Cr (III), Cd (II) and Mn (II( by the bacterial biomass from 3,311.78 cm^− 1^ to 3,292.49 cm^− 1^, this peak is caused by the presence of hydroxyl (-OH) and amino groups (-NH), which are essential for hydrogen bonding and water structuring and binding proteins together, the peak is observed to be shifted after biosorption of the metal ions by the bacterial biomass demonstrating the groups’ involvement in the biosorption process, this explanation have been well-documented in several researches^57,63^. The recent investigation revealed that N–H and C–H stretching may be attributed to the bands in the ranges of 2800–3000 cm^− 1^ and 2900–2970 cm^− 1^, respectively; these groups belong to the protein, carbohydrates, and other substances in the bacterial cell wall, similar results were obtained by^57,58,63^. In our FTIR spectra, the absorption peak of the carboxylic acid (C = O) peak at 1,647.21 cm^− 1^was widening after adsorption; the peak moved to 1645.28 in biomass loaded with chromium; significant variations in the wave number indicate that the carboxylic acid is involved in the process of ion exchange. The absorption peak at 1448.54 cm^− 1^ attributed to N-H vibrations, the peak moved to 1489.05 cm-1 in biomass loaded with Manganese. The absorption peak of the phosphoryl (P = O stretching vibration) at 1,404 cm^− 1^ was weaker than before adsorption and the absorption peak moved to 1,400 cm^− 1^ in bacteria loaded with Cadmium, Chromium and Manganese. The absorption peak at 1,238 cm^− 1^ which caused by the asymmetric stretching vibration of P = O of the phosphate group moved to 1,242 cm^− 1^, the peak become weaker in biomass loaded with Chromium and Manganese, and also the absorption peak become widening in biomass loaded with Cadmium. The absorption peak at 1,072 cm^− 1^ is caused by P = O symmetric stretching vibration and the absorption peaks were widening, also the position moved after adsorption to 1,068 cm^− 1^ in biomass loaded with cadmium and manganese, and moved to 1,049 cm^− 1^ in biomass loaded with chromium. Furthermore, the IR spectra of bacterial biomass indicated an absorption band at 532.35 cm^− 1^ due to a strong stretching vibration of halogen linked to the ligand moved to 536.21 cm^− 1^ & 538.14 cm^− 1^ in biomass loaded by cadmium and manganese respectively. In the current investigation Cr, Cd, and Mn ions were confirmed to be removed from the aqueous solution by the shifting of the peaks associated with functional groups, such as hydroxyl, amines, phosphoryl, and carbonyl, which indicate an interaction between metals and the biomass functional groups. Therefore, The FTIR results suggest that the *Staphylococcus devriesei* strain MS is useful for the biosorption of Cr, Cd, and Mn ions. FTIR analysis of the studied isolate confirms that the functional groups involved in the biosorption process and metal removal may have hydroxyl, alkyl, amide, halogen, phosphoryl, and phosphoric acid groups.

The characterization of bacterial biomass by scanning electron microscopy (SEM) provides elemental and topographical data of the solids with a virtually enormous depth of field, allowing various specimen parts to keep in focus at a time. High resolution in SEM also enables greater magnification for closely spaced materials. In addition to its capability to create an actual clear image, it is also helpful for discovering the topographical aspects of bacterial biomass before and after metal loading^47^. In the current study the EDX analysis was used to determine the insertion of the studied metal ions (Cd, Cr, and Mn) into the cell wall of *Staphylococcus devriesei* strain MS after the biosorption process. In the bacterial cell, biosorption was observed on the surface of cell wall that contains the functional group such as sulfate, carboxyl, and hydroxyl. The variation in the biosorption of Cr (III), Cd (II), and Mn (II) were due to the different affinities of the metallic ions for the functional groups of the polysaccharides in the cellular walls of bacterial cell. In our study, the normal bacterial cell has smooth round shape and normal appearance^62^. After Biosorption process, the cell wall, shape, and size of the bacteria changed. Depicted data as shown in Figs. 2, 3, 4, 5, 6, 7, and 8 illustrated that the bacterial cell wall after biosorption process to shrink and stick. Also, the cell wall became non smooth, irregular, deposits on the cell surface and had many depressions on them. EDX analysis demonstrated the presence of extra peaks after the biosorption process that belongs to the absorbed metal ions. In the current investigation, after biosorption of Cr (III) the bacterial cells became rough, irregular and were cracked with the appearance of wrinkles on its surface, also SEM pictures revealed that chromium was uniformly bound on the cell wall surface of the bacteria^46,60^, EDX analysis showed the insertion of Cr (III). Also, after biosorption of Cd (II) the bacterial cells were smaller in size and cracked, cell surface became rough, wrinkled and porous, the results have great agreement with^64^. Strong crosslinking of the metal (Cd) and negatively charged chemical groups on the cell wall polymers was the cause of this structural alteration, similar results were described by^46,62,65^. EDX analysis demonstrated the insertion of Cd (II) into the bacterial biomass. Also, after biosorption of metal ions, there are many flocs on the surface of the strain. After biosorption of Mn (II), the bacterial cells became brilliant, had smaller size, and the bacterial cells became contiguous and these results supported the results of^51,65^. In addition, EDX analysis showed the presence of Mn (II). The change in the morphology showed the addition of concentration of charged moieties of the metal ions onto surface areas of the bacterial biomass and the pores that found on the bacterial cell wall facilitated the opportunity for metal ions biosorption. In the biosorption process occurred on the surface of cell wall of the bacterial cells that contains the functional group such as hydroxyl, alkyl, amide, sulfoxide, and phosphoryl groups, the variation in the biosorption of Cr (III), Cd (II), and Mn (II) were due to affinities of metal ions for the functional groups of the polysaccharides in the cellular walls of bacterial biomass. The results related to the application of *Staphylococcus devriesei* in drinking water sources (raw water) in Fayoum Governorate, Egypt demonstrated the effective role of the studied bacteria in the metal removal process and the applicability of the present biosorption method. It was observed that the rate of removal of heavy metals in water is higher than the application under controlled laboratory conditions. Our explanation for this is due to the presence of a microbial community that is likely to work together for the best removal and best work. *Staphylococcus devriesei* strain MS as biosorbent is useful in metal removal due to low cost, easy availability, and high affinity for metal ions.

**Fig. 14:**
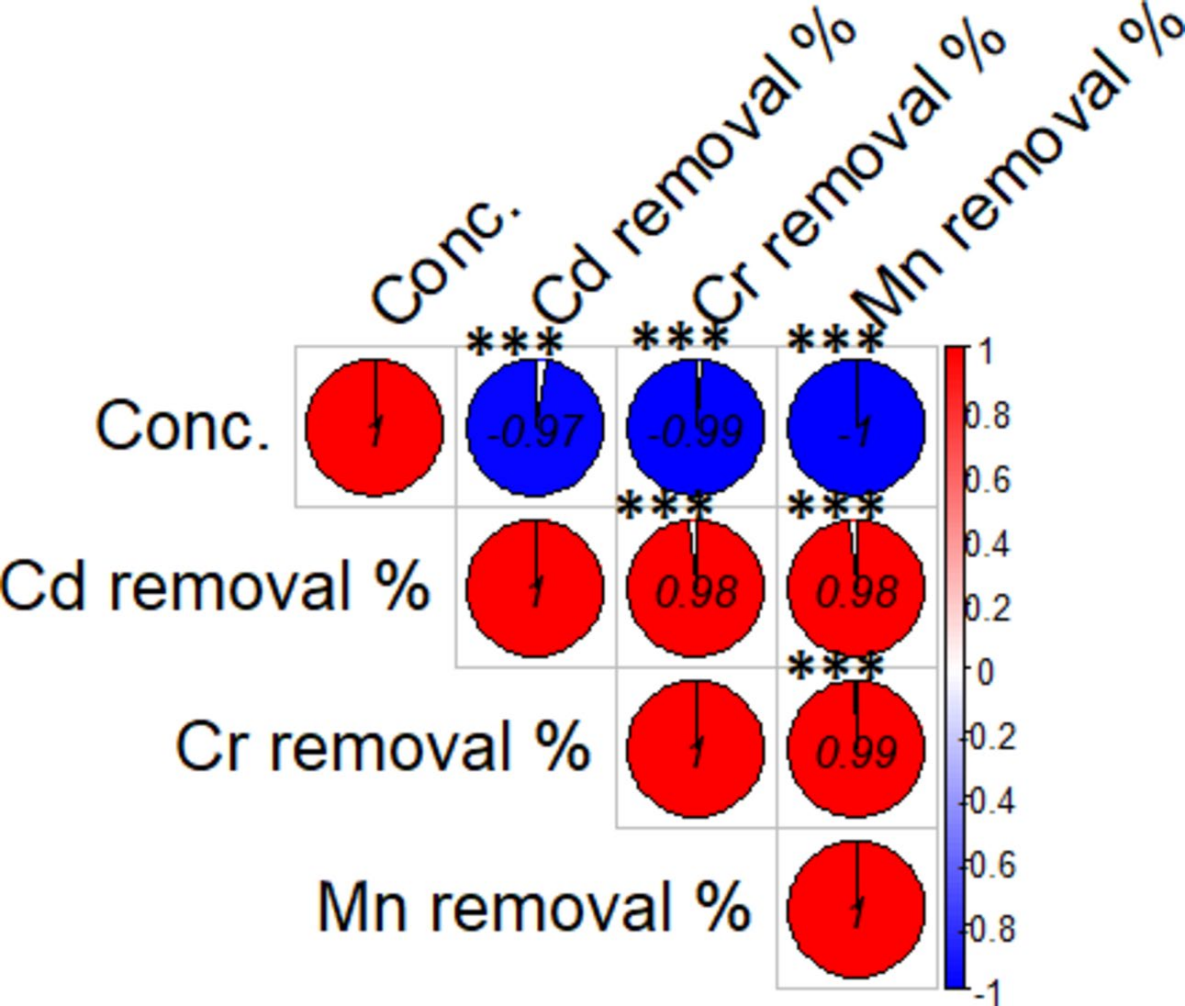
The correlations between metal ion concentration and the metal removal.

## Conclusion

According to the findings, *S. devriesei* MS has a great deal of potential for use in applications that remove moderate to light amounts of Cd, Cr, and Mn from contaminated water (under controlled conditions and raw drinking water in Fayoum, Egypt) in an efficient and cost-effective manner. SEM was used to detect the morphological changes of the bacterial cells that were based on the type of the metal. When comparing the metal treatment samples to the control, the elemental EDX analysis verified the presence of additional identifiable metals. The bacterial cells’ ultrastructure showed that the heavy metals were deposited on the cell walls as well as inside and outside of the cells.

## Data Availability

All data generated or analyzed during this study are included in this published article.
